# Supplementary material for: Molecular Environment Effects That Modulate the Photophysical Properties of Novel 1,3-Phosphinoamines Based on 2,1,3-Benzothiadiazole
Source: Molecules. 2022 Jun 16;27(12):3857. doi: 10.3390/molecules27123857 (PMC9227927; doi:10.3390/molecules27123857)
Supplement: Supplementary file 1 [file molecules-27-03857-s001.zip › molecules-1761055-supplementary.pdf]

Supporting information

Molecular environment effects that modulate the photophysical properties of novel 1,3-phosphinoamines based on 2,1,3-benzothiadiazole

Radmir M. Khisamov, Alexey A. Ryadun, Sergey N. Konchenko, Taisiya S. Sukhikh

IR-spectra of the compounds 1
Crystal data and structure refinement 2
Powder X-ray diffraction data 3
Photographs of crystals 2α and 2β·thf under visible and UV light 5
Geometry and energy features of the molecules 6
DFT calculation of Frank-Condon excitation processes 7
Frontier orbitals 17
DFT calculation of relaxation processes 25
Photoluminescence spectra 27

IR-spectra of the compounds

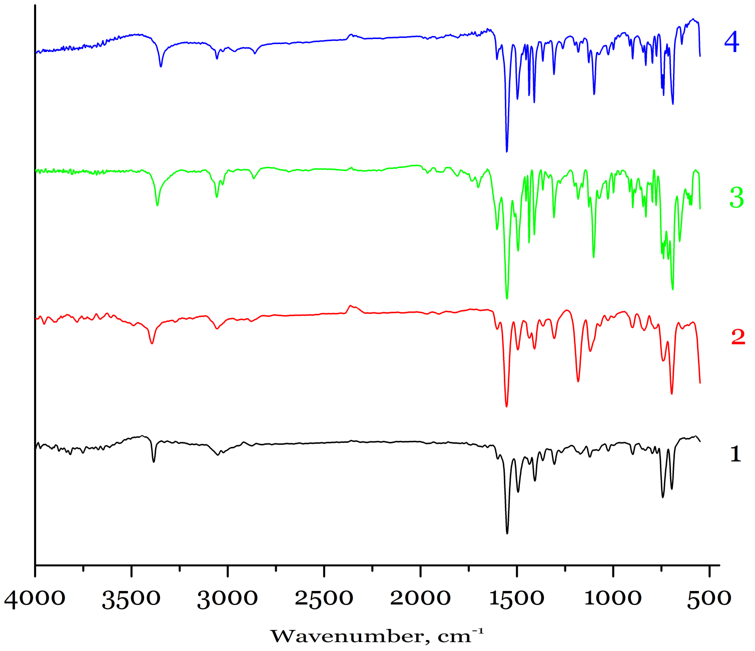

Figure S1. IR-spectra of compounds **1**–**4** recorded in KBr pellets.

Table S1. Crystal data and structure refinement for the compounds

Identification code	**1**	**2α**	**2β**·thf	**3**	**4**
Empirical formula	$C_{25}H_{20}N_3PS$	$C_{25}H_{20}N_3OPS$	$C_{29}H_{28}N_3O_2PS$	$C_{25}H_{20}N_3PS_2$	$C_{25}H_{20}N_3PSSe$
Formula weight	425.47	441.47	513.57	457.53	504.43
Temperature/K	150(2)	240(2)	150(2)	150(2)	150(2)
Space group	*P*–1	$P2_1$	*Pbca*	$P2_1/n$	$P2_1/n$
a/Å	9.335(3)	5.8869(5)	10.9504(16)	9.2084(4)	9.1686(8)
b/Å	10.765(4)	19.2234(12)	17.815(3)	20.3488(10)	20.5591(17)
c/Å	12.793(5)	9.7889(10)	27.605(3)	12.6978(5)	12.7179(9)
α/°	65.636(11)	90	90	90	90
β/°	82.580(12)	104.521(4)	90	109.844(2)	109.002(2)
γ/°	65.493(12)	90	90	90	90
Volume/Å^3	1064.2(7)	1072.39(16)	5385.1(13)	2238.03(17)	2266.7(3)
Z	2	2	8	4	4
ρ_{calc}g/cm^3	1.328	1.367	1.267	1.358	1.478
μ/mm^{-1}	0.244	0.249	0.210	0.328	1.837
F(000)	444.0	460.0	2160.0	952.0	1024.0
Radiation	MoKα (λ = 0.71073)	MoKα (λ = 0.71073)	MoKα (λ = 0.71073)	MoKα (λ = 0.71073)	MoKα (λ = 0.71073)
2Θ range for data collection/°	4.492 to 48.846	4.238 to 53.024	2.95 to 48.898	4.004 to 54.246	4.816 to 52.764
Index ranges	-10 ≤ h ≤ 10, -12 ≤ k ≤ 12, -13 ≤ l ≤ 14	-5 ≤ h ≤ 7, -24 ≤ k ≤ 17, -12 ≤ l ≤ 8	-12 ≤ h ≤ 12, -20 ≤ k ≤ 20, -32 ≤ l ≤ 32	-10 ≤ h ≤ 11, -26 ≤ k ≤ 26, -16 ≤ l ≤ 12	-11 ≤ h ≤ 11, -25 ≤ k ≤ 25, -15 ≤ l ≤ 15
Reflections collected	7491	5271	39661	31244	25940
Independent reflections	3424 [R_{int} = 0.0824, R_{sigma} = 0.1288]	3627 [R_{int} = 0.0255, R_{sigma} = 0.0598]	4449 [R_{int} = 0.1484, R_{sigma} = 0.0888]	4923 [R_{int} = 0.0394, R_{sigma} = 0.0282]	4650 [R_{int} = 0.0598, R_{sigma} = 0.0413]
Data/restraints/parameters	3424/1/274	3627/2/283	4449/10/300	4923/1/283	4650/1/283
Goodness-of-fit on F^2	1.037	1.019	1.054	1.023	1.046
Final R indexes [I>=2σ (I)]	R_1 = 0.0664, wR_2 = 0.1537	R_1 = 0.0406, wR_2 = 0.0759	R_1 = 0.0882, wR_2 = 0.2463	R_1 = 0.0437, wR_2 = 0.1066	R_1 = 0.0342, wR_2 = 0.0798
Final R indexes [all data]	R_1 = 0.0964, wR_2 = 0.1825	R_1 = 0.0542, wR_2 = 0.0821	R_1 = 0.1352, wR_2 = 0.2907	R_1 = 0.0533, wR_2 = 0.1131	R_1 = 0.0452, wR_2 = 0.0881
Largest diff. peak/hole / e Å^{-3}	0.66/-0.41	0.20/-0.24	1.00/-0.59	1.42/-0.41	0.57/-0.33
Flack parameter		0.04(7)			

Powder X-ray diffraction data

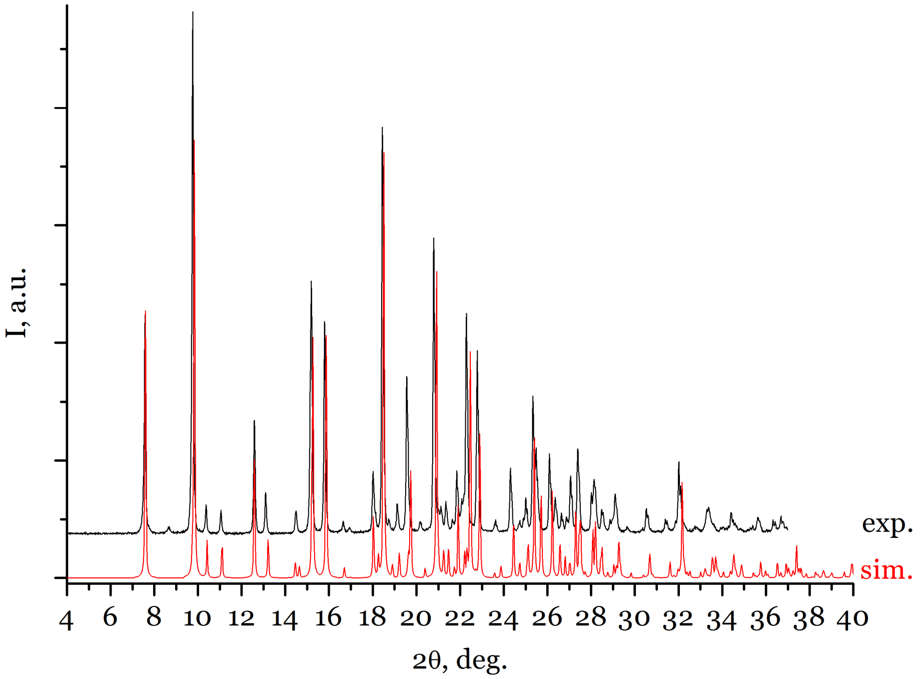

Figure S2. Simulated and experimental powder XRD patterns for compound **1** (Cu K_α radiation). Minor peak at 8.68° does not correspond to any of phase of oxidized species **2**.

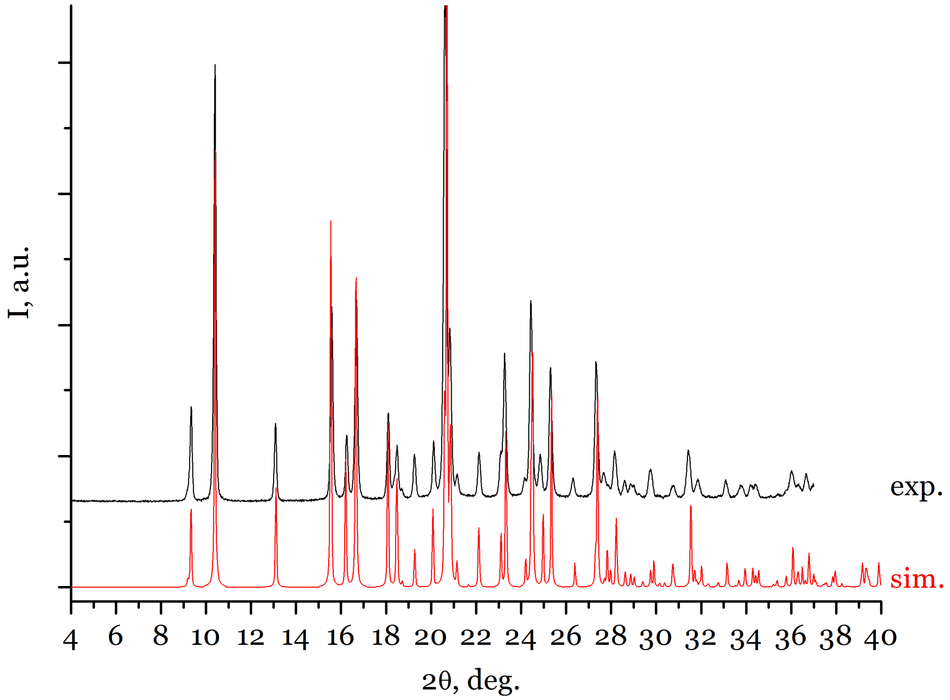

Figure S3. Simulated and experimental powder XRD patterns for compound **2α** (Cu K_α radiation).

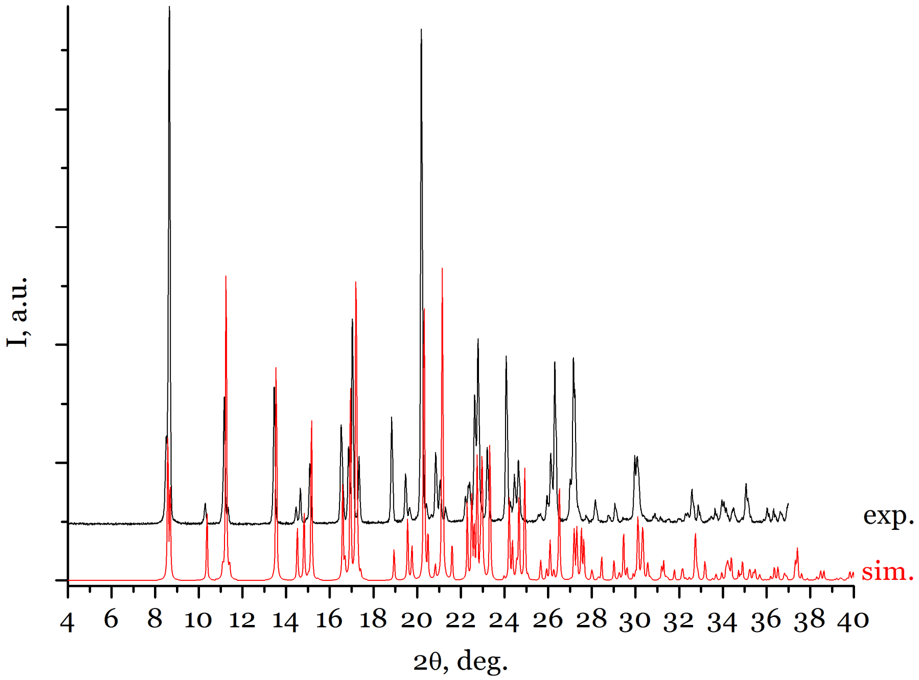

Figure S4. Simulated and experimental powder XRD patterns for compound **3** (Cu K_α radiation).

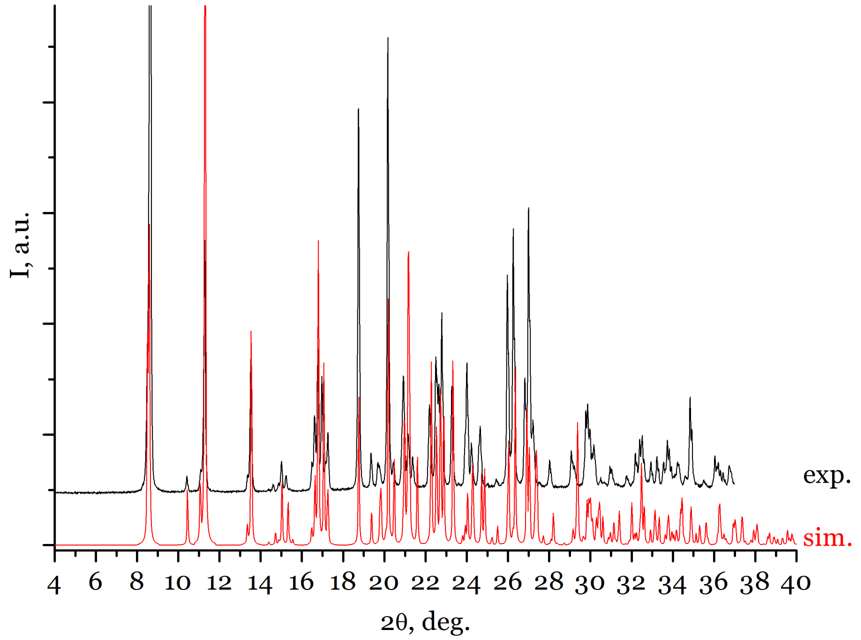

Figure S5. Simulated and experimental powder XRD patterns for compound **4** (Cu K_α radiation).

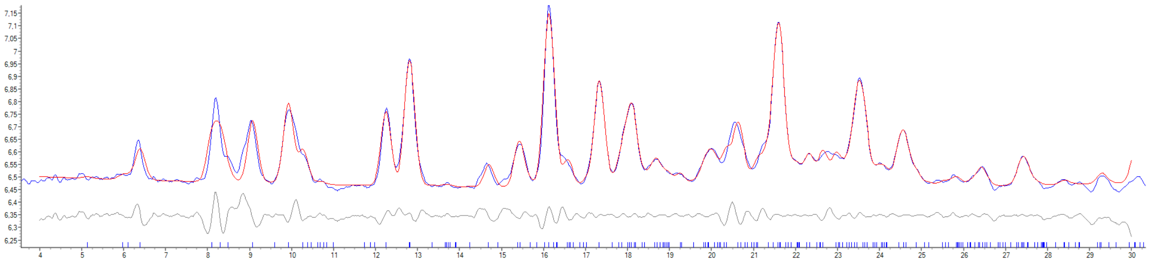

Figure S6. Pawley refinement for the powder pattern for the solvate-free phase, **2β** (Cu K_{α} radiation, 150K). Refined unit cell parameters: monoclinic *P*, *a* = 17.21 Å, *b* = 10.41 Å, *a* = 27.64 Å, β = 91.43°, V = 4952.6 Å^3. The fit at low angles is not good, probably because of the presence of a number of related phases.

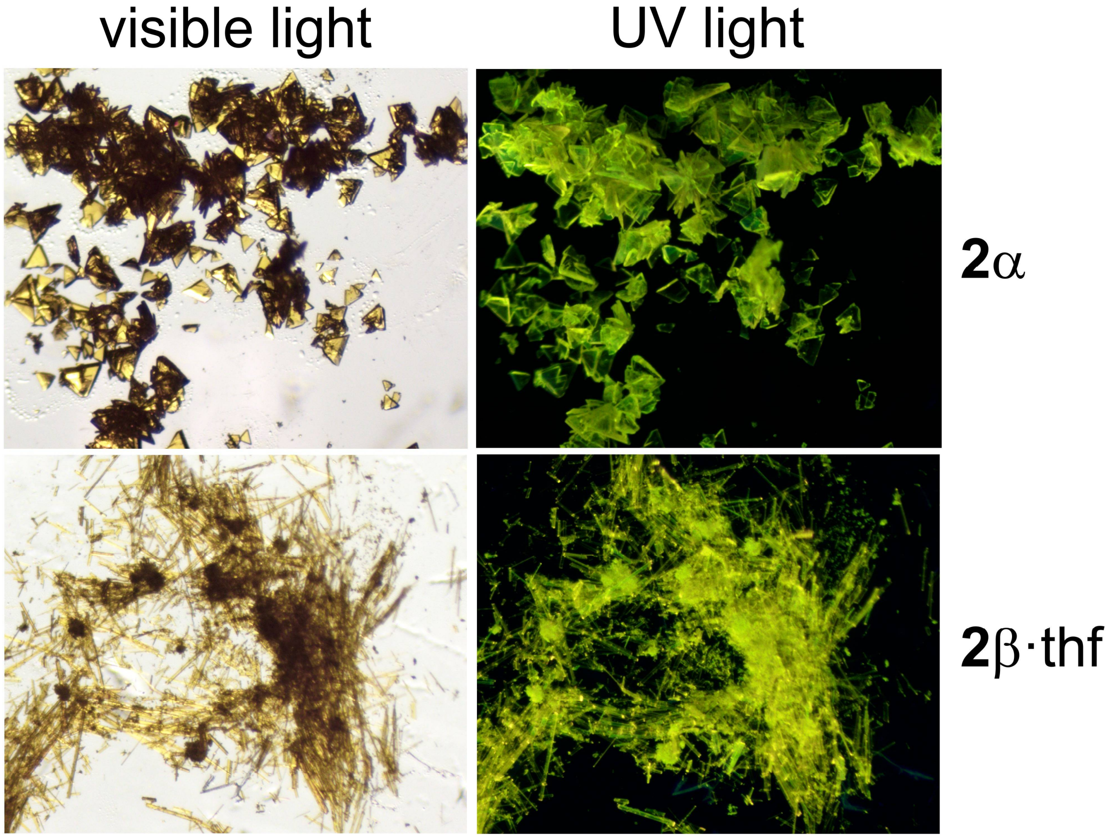

Figure S7. Photographs of crystals **2α** and **2β**·thf under visible and UV light.

Geometry and energy features of the molecules of the compounds

Table S2. Estimated contributions to the interaction energy (kJ/mol) between a couple of the molecules in **2α** and **2β**·THF, calculated in the CrystalExplorer v. 17.5 program. Level of theory B3LYP/6-31G(d,p).

	electrostatic	polarization	dispersion	repulsion	total
**2α**	-22.4	-8.3	-68.8	46.3	-61.2
**2β**·THF	-51.3	-17.0	-83.4	87.3	-85.6

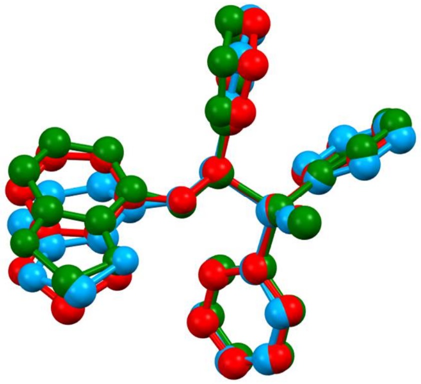

Figure S8. Overlay of the molecules of structures **2α** (red), **2β**·thf (blue), and **3** and **4** (green) on the example of the latter.

DFT calculation of Frank-Condon excitation processes

Table S3. TD-DFT calculated excitation transitions of **1** in the ground state (PBE0/def2-TZVP(-f)). H – HOMO, L – LUMO

State	Energy, cm^{-1}	Wavelength, nm	Oscilator strength	Transitions	Contributions
1	23603.2	423.7	0.088385263	H → L	0.983184
2	30858.3	324.1	0.000466761	H - 1 → L	0.992984
3	33796.6	295.9	0.035534635	H - 1 → L + 1<br>H → L + 1	0.013506<br>0.964632
4	34724.2	288.0	0.004914953	H → L + 2<br>H → L + 4	0.956257<br>0.012231
5	35163.6	284.4	0.008651734	H - 2 → L	0.973192
6	36289.2	275.6	0.010554810	H - 4 → L<br>H → L + 3<br>H → L + 4	0.010929<br>0.928550<br>0.018669
7	36910.7	270.9	0.030703125	H - 5 → L<br>H - 4 → L<br>H - 3 → L<br>H → L + 3<br>H → L + 4<br>H → L + 5<br>H → L + 6<br>H → L + 7	0.026211<br>0.374705<br>0.059173<br>0.038135<br>0.287557<br>0.021079<br>0.016580<br>0.116321
8	37515.1	266.6	0.001407458	H - 4 → L<br>H - 3 → L<br>H → L + 4	0.012650<br>0.901086<br>0.069235
9	37713.9	265.2	0.059277820	H - 4 → L<br>H - 3 → L<br>H - 1 → L + 1<br>H → L + 4<br>H → L + 5<br>H → L + 7	0.259063<br>0.025129<br>0.092984<br>0.478530<br>0.011550<br>0.077540
10	37918.9	263.7	0.056846632	H - 4 → L<br>H - 1 → L + 1<br>H - 1 → L + 2<br>H → L + 1<br>H → L + 4<br>H → L + 5	0.016085<br>0.781297<br>0.011558<br>0.013295<br>0.036223<br>0.083727
11	38288.1	261.2	0.005869281	H - 1 → L + 1<br>H → L + 4<br>H → L + 5<br>H → L + 6	0.055339<br>0.062663<br>0.762327<br>0.092988
12	38355.0	260.7	0.001843530	H - 5 → L<br>H - 4 → L	0.900025<br>0.071466

13	39113.8	255.7	0.025258606	H - 6 → L<br>H - 4 → L<br>H - 1 → L + 2<br>H - 1 → L + 3<br>H → L + 4<br>H → L + 5<br>H → L + 6	0.024238<br>0.014336<br>0.268146<br>0.054225<br>0.015528<br>0.028875<br>0.511625
14	39349.3	254.1	0.017835553	H - 10 → L<br>H - 6 → L<br>H - 5 → L + 1<br>H - 1 → L + 2<br>H - 1 → L + 3<br>H - 1 → L + 4<br>H → L + 5<br>H → L + 6	0.165630<br>0.121298<br>0.010677<br>0.399077<br>0.040229<br>0.013603<br>0.027725<br>0.143151
15	39382.3	253.9	0.012700551	H - 10 → L<br>H - 7 → L<br>H - 6 → L<br>H - 1 → L + 2<br>H → L + 5<br>H → L + 6	0.360734<br>0.017900<br>0.264096<br>0.101952<br>0.027357<br>0.183191
16	39881.5	250.7	0.001537155	H - 10 → L<br>H - 9 → L<br>H - 7 → L<br>H - 6 → L<br>H - 5 → L	0.333066<br>0.010557<br>0.077524<br>0.546282<br>0.010972
17	40449.2	247.2	0.000581606	H - 10 → L<br>H - 7 → L<br>H - 6 → L	0.093680<br>0.885331<br>0.012305
18	40994.4	243.9	0.109312117	H - 5 → L + 1<br>H - 5 → L + 2<br>H - 1 → L + 1<br>H - 1 → L + 2<br>H - 1 → L + 3<br>H - 1 → L + 4<br>H - 1 → L + 5<br>H - 1 → L + 6	0.038261<br>0.021754<br>0.011175<br>0.138123<br>0.574798<br>0.084399<br>0.019592<br>0.020918
19	41755.9	239.5	0.021142081	H - 7 → L + 1<br>H - 7 → L + 3<br>H - 6 → L + 1<br>H - 5 → L + 2<br>H - 5 → L + 3<br>H - 3 → L + 1<br>H - 2 → L + 2<br>H - 1 → L + 3<br>H - 1 → L + 4	0.017262<br>0.016547<br>0.028102<br>0.027351<br>0.027088<br>0.016558<br>0.019060<br>0.160150<br>0.386272

				H - 1 → L + 5<br>H - 1 → L + 6<br>H → L + 6	0.032427<br>0.156600<br>0.020728
20	42607.8	234.7	0.006812253	H - 6 → L + 1<br>H - 6 → L + 2<br>H - 5 → L + 2<br>H - 3 → L + 2<br>H - 3 → L + 3<br>H - 3 → L + 4<br>H - 3 → L + 5<br>H - 2 → L + 1<br>H - 2 → L + 2<br>H - 2 → L + 4<br>H - 2 → L + 5<br>H - 2 → L + 6<br>H - 1 → L + 4<br>H - 1 → L + 5<br>H - 1 → L + 6	0.027828<br>0.010721<br>0.012360<br>0.034191<br>0.016479<br>0.056655<br>0.050855<br>0.020480<br>0.109913<br>0.063766<br>0.064120<br>0.075291<br>0.074597<br>0.260422<br>0.027229

Table S4. TD-DFT calculated excitation transitions of **2** in the ground state (PBE0/def2-TZVP(-f)). H – HOMO, L – LUMO

State	Energy, cm-1	Wavelength, nm	Oscilator strength	Transitions	Contributions
1	24452.0	409.0	0.093202804	H → L	0.982907
2	31060.0	322.0	0.006405624	H → L + 1<br>H → L + 2	0.971445<br>0.017801
3	33346.9	299.9	0.007754882	H → L + 1<br>H → L + 2	0.019074<br>0.956273
4	35724.0	279.9	0.007844019	H - 2 → L<br>H - 1 → L<br>H → L + 3<br>H → L + 5<br>H → L + 7	0.028463<br>0.099713<br>0.673194<br>0.171264<br>0.011693
5	36114.9	276.9	0.013274467	H - 1 → L<br>H → L + 3<br>H → L + 4<br>H → L + 5<br>H → L + 7	0.661009<br>0.191622<br>0.100547<br>0.019872<br>0.012689
6	36290.6	275.6	0.002032503	H - 1 → L<br>H → L + 4<br>H → L + 5<br>H → L + 6	0.179554<br>0.597541<br>0.150762<br>0.051341
7	36969.6	270.5	0.009086558	H - 2 → L<br>H - 1 → L<br>H → L + 2	0.160250<br>0.036519<br>0.011967

				H → L + 3<br>H → L + 4<br>H → L + 5<br>H → L + 6<br>H → L + 7	0.084790<br>0.053615<br>0.224108<br>0.384419<br>0.029793
8	37694.1	265.3	0.033027295	H - 2 → L<br>H → L + 3<br>H → L + 4<br>H → L + 6<br>H → L + 7	0.289729<br>0.014242<br>0.104340<br>0.468185<br>0.091520
9	37774.5	264.7	0.068130443	H - 2 → L<br>H → L + 3<br>H → L + 4<br>H → L + 5<br>H → L + 6<br>H → L + 7	0.264653<br>0.022543<br>0.135114<br>0.407429<br>0.077671<br>0.064229
10	39266.7	254.7	0.000059605	H - 11 → L<br>H - 3 → L	0.012242<br>0.967068
11	39582.0	252.6	0.000040603	H - 11 → L<br>H - 3 → L	0.961607<br>0.012033
12	40227.2	248.6	0.033392282	H - 2 → L + 1<br>H - 1 → L + 1<br>H - 1 → L + 2	0.017067<br>0.828545<br>0.097714
13	41843.3	239.0	0.006499257	H - 12 → L<br>H - 9 → L<br>H - 8 → L<br>H - 7 → L<br>H - 6 → L<br>H - 5 → L<br>H - 4 → L	0.017433<br>0.016890<br>0.050853<br>0.052278<br>0.014216<br>0.406137<br>0.405517
14	42224.0	236.8	0.051539131	H - 6 → L + 2<br>H - 5 → L<br>H - 5 → L + 1<br>H - 5 → L + 3<br>H - 5 → L + 4<br>H - 4 → L + 1<br>H - 4 → L + 2<br>H - 3 → L + 1<br>H - 3 → L + 4<br>H - 3 → L + 5<br>H - 2 → L + 2<br>H - 1 → L + 1<br>H - 1 → L + 2<br>H - 1 → L + 3<br>H - 1 → L + 6	0.010922<br>0.017765<br>0.033105<br>0.010007<br>0.017201<br>0.057468<br>0.028052<br>0.053709<br>0.012754<br>0.028906<br>0.013208<br>0.089189<br>0.441628<br>0.010315<br>0.050115

15	42401.5	235.8	0.001864814	H - 8 → L<br>H - 7 → L + 3<br>H - 6 → L<br>H - 6 → L + 1<br>H - 6 → L + 2<br>H - 5 → L<br>H - 5 → L + 1<br>H - 4 → L<br>H - 1 → L + 2	0.031405<br>0.018270<br>0.326099<br>0.038957<br>0.012509<br>0.246126<br>0.011180<br>0.228547<br>0.029961
16	42579.0	234.9	0.001808278	H - 6 → L<br>H - 6 → L + 1<br>H - 5 → L + 1<br>H - 5 → L + 3<br>H - 5 → L + 4<br>H - 4 → L + 1<br>H - 4 → L + 2<br>H - 4 → L + 3<br>H - 3 → L + 1<br>H - 3 → L + 5<br>H - 1 → L + 3<br>H - 1 → L + 4<br>H - 1 → L + 6	0.015125<br>0.011058<br>0.121507<br>0.021788<br>0.048464<br>0.061933<br>0.092235<br>0.023525<br>0.091093<br>0.095689<br>0.052809<br>0.117956<br>0.121427
17	42777.7	233.8	0.003880932	H - 8 → L<br>H - 7 → L<br>H - 7 → L + 3<br>H - 7 → L + 4<br>H - 6 → L<br>H - 6 → L + 1<br>H - 6 → L + 2<br>H - 5 → L<br>H - 4 → L<br>H - 1 → L + 2<br>H - 1 → L + 3	0.025998<br>0.023336<br>0.036618<br>0.016922<br>0.266538<br>0.102661<br>0.024710<br>0.170073<br>0.257313<br>0.013434<br>0.011986
18	43093.5	232.1	0.014453605	H - 7 → L<br>H - 7 → L + 3<br>H - 6 → L<br>H - 6 → L + 1<br>H - 6 → L + 2<br>H - 5 → L + 1<br>H - 4 → L<br>H - 4 → L + 1<br>H - 4 → L + 2<br>H - 3 → L + 1<br>H - 3 → L + 5<br>H - 1 → L + 1<br>H - 1 → L + 2	0.019873<br>0.032218<br>0.212270<br>0.062859<br>0.027923<br>0.067911<br>0.018446<br>0.032764<br>0.016972<br>0.056524<br>0.021038<br>0.010401<br>0.205101

				H - 1 → L + 4<br>H - 1 → L + 6	0.017128<br>0.064108
19	43204.2	231.5	0.028836467	H - 8 → L<br>H - 7 → L<br>H - 5 → L<br>H - 5 → L + 1<br>H - 4 → L<br>H - 4 → L + 2<br>H - 1 → L + 2<br>H - 1 → L + 6<br>H → L + 7	0.145517<br>0.591789<br>0.044172<br>0.023821<br>0.045320<br>0.012190<br>0.039296<br>0.013322<br>0.019673
20	43307.5	230.9	0.024382549	H - 8 → L<br>H - 7 → L<br>H - 7 → L + 2<br>H - 7 → L + 3<br>H - 7 → L + 4<br>H - 6 → L<br>H - 6 → L + 1<br>H - 6 → L + 2<br>H - 5 → L<br>H - 5 → L + 4<br>H - 4 → L + 2<br>H - 4 → L + 3<br>H - 3 → L + 1<br>H - 3 → L + 5<br>H - 1 → L + 2<br>H - 1 → L + 3<br>H - 1 → L + 6	0.017834<br>0.058846<br>0.013126<br>0.052431<br>0.032835<br>0.148146<br>0.238928<br>0.030675<br>0.011944<br>0.025775<br>0.040183<br>0.020897<br>0.031572<br>0.014107<br>0.086873<br>0.017083<br>0.057765

Table S5. TD-DFT calculated excitation transitions of **3** in the ground state (PBE0/def2-TZVP(-f)). H – HOMO, L – LUMO

State	Energy, cm-1	Wavelength, nm	Oscilator strength	Transitions	Contributions
1	24646.6	405.7	0.098596968	H → L	0.982543
2	31554.9	316.9	0.000122585	H - 1 → L	0.986023
3	32253.6	310.0	0.000546470	H - 2 → L	0.986274
4	32522.0	307.5	0.001654765	H - 1 → L + 2<br>H → L + 1<br>H → L + 2	0.011608<br>0.294326<br>0.672823
5	32900.2	303.9	0.000562491	H → L + 1<br>H → L + 2	0.679752<br>0.293760
6	35354.6	282.8	0.009862619	H - 3 → L<br>H - 1 → L + 2<br>H → L + 3	0.010171<br>0.050954<br>0.395442

				H → L + 4<br>H → L + 5	0.444448<br>0.070859
7	35644.4	280.5	0.018136818	H - 2 → L + 1<br>H - 1 → L + 1<br>H - 1 → L + 2<br>H → L + 4	0.209668<br>0.663920<br>0.068698<br>0.024790
8	35992.4	277.8	0.028033330	H - 2 → L + 1<br>H - 1 → L + 1<br>H - 1 → L + 2<br>H → L + 2<br>H → L + 4<br>H → L + 5	0.015232<br>0.081421<br>0.778714<br>0.015037<br>0.060330<br>0.013679
9	36186.6	276.3	0.014274214	H - 3 → L<br>H - 2 → L + 1<br>H - 2 → L + 2<br>H - 1 → L + 1<br>H - 1 → L + 2<br>H → L + 3<br>H → L + 4<br>H → L + 5<br>H → L + 6	0.035205<br>0.089773<br>0.014671<br>0.015313<br>0.036172<br>0.492297<br>0.213113<br>0.057185<br>0.012094
10	36373.6	274.9	0.011632147	H - 2 → L + 1<br>H - 2 → L + 2<br>H - 1 → L + 1<br>H - 1 → L + 2<br>H → L + 1<br>H → L + 3<br>H → L + 4	0.478027<br>0.207960<br>0.154769<br>0.013350<br>0.011827<br>0.073947<br>0.018826
11	36898.6	271.0	0.078599657	H - 4 → L<br>H - 3 → L<br>H → L + 4<br>H → L + 5<br>H → L + 6<br>H → L + 7	0.039938<br>0.672184<br>0.101538<br>0.033186<br>0.019595<br>0.086655
12	37231.2	268.6	0.002774709	H - 2 → L + 1<br>H - 2 → L + 2<br>H - 1 → L + 1	0.174827<br>0.725451<br>0.046854
13	37494.7	266.7	0.006782619	H - 4 → L<br>H - 3 → L<br>H → L + 4<br>H → L + 5<br>H → L + 6	0.075433<br>0.156865<br>0.061434<br>0.557764<br>0.127539
14	37884.5	264.0	0.044685130	H - 5 → L<br>H - 4 → L<br>H - 3 → L<br>H → L + 5	0.045070<br>0.616992<br>0.057207<br>0.118031

				H → L + 6<br>H → L + 7	0.027568<br>0.102992
15	38736.1	258.2	0.000481069	H - 5 → L<br>H - 4 → L	0.895485<br>0.091165
16	39238.5	254.9	0.001880633	H - 1 → L + 3<br>H → L + 4<br>H → L + 5<br>H → L + 6	0.368365<br>0.034522<br>0.075280<br>0.479065
17	39288.6	254.5	0.005875222	H - 8 → L + 2<br>H - 2 → L + 3<br>H - 1 → L + 3<br>H - 1 → L + 4<br>H → L + 3<br>H → L + 4<br>H → L + 5<br>H → L + 6	0.011845<br>0.028120<br>0.481911<br>0.058764<br>0.010570<br>0.024202<br>0.041425<br>0.307038
18	39424.2	253.7	0.000052775	H - 11 → L	0.974360
19	40302.9	248.1	0.000261880	H - 2 → L + 3<br>H - 2 → L + 4<br>H - 2 → L + 5<br>H - 1 → L + 4	0.600364<br>0.039055<br>0.018596<br>0.286421
20	40753.2	245.4	0.011202698	H - 2 → L + 3<br>H - 2 → L + 4<br>H - 1 → L + 3<br>H - 1 → L + 4<br>H - 1 → L + 5<br>H → L + 6	0.216165<br>0.046586<br>0.067204<br>0.561989<br>0.023421<br>0.011996

Table S6. TD-DFT calculated excitation transitions of **4** in the ground state (PBE0/def2-TZVP(-f)). H – HOMO, L – LUMO

State	Energy, cm-1	Wavelength, nm	Oscilator strength	Transitions	Contributions
1	24616.8	406.2	0.100692050	H → L	0.972388
2	28567.4	350.0	0.000526958	H - 1 → L<br>H → L	0.983238<br>0.010709
3	29466.9	339.4	0.000416303	H - 2 → L	0.990800
4	31169.6	320.8	0.012446356	H - 2 → L + 1<br>H - 2 → L + 2<br>H - 1 → L + 1<br>H - 1 → L + 2<br>H → L + 1<br>H → L + 2	0.020405<br>0.011318<br>0.173446<br>0.029861<br>0.601190<br>0.145299
5	31854.6	313.9	0.001087329	H - 1 → L + 1<br>H - 1 → L + 2	0.043821<br>0.216688

				H → L + 1<br>H → L + 2	0.136232<br>0.586290
6	32338.8	309.2	0.014399165	H - 2 → L + 1<br>H - 1 → L + 1<br>H → L + 1	0.335701<br>0.548007<br>0.090553
7	33188.7	301.3	0.016821731	H - 2 → L + 1<br>H - 2 → L + 2<br>H - 1 → L + 2<br>H → L + 2	0.023915<br>0.070631<br>0.667460<br>0.204291
8	33464.9	298.8	0.006257015	H - 2 → L + 1<br>H - 2 → L + 2<br>H - 1 → L + 1<br>H → L + 1	0.551306<br>0.087261<br>0.177631<br>0.154873
9	34466.2	290.1	0.003331934	H - 2 → L + 1<br>H - 2 → L + 2<br>H - 1 → L + 1<br>H - 1 → L + 2<br>H → L + 2<br>H → L + 3	0.037685<br>0.791690<br>0.025540<br>0.050494<br>0.055131<br>0.013425
10	34892.3	286.6	0.014619474	H - 1 → L + 3<br>H → L + 3<br>H → L + 4<br>H → L + 5	0.022564<br>0.467376<br>0.410051<br>0.052081
11	35650.5	280.5	0.008166630	H - 3 → L<br>H - 2 → L + 2<br>H - 1 → L + 3<br>H → L + 3<br>H → L + 4<br>H → L + 5<br>H → L + 6	0.011456<br>0.010067<br>0.124800<br>0.355467<br>0.396093<br>0.067647<br>0.010082
12	36532.4	273.7	0.004617130	H - 2 → L + 3<br>H - 1 → L + 3<br>H - 1 → L + 4<br>H → L + 3<br>H → L + 4	0.050978<br>0.743305<br>0.034330<br>0.114785<br>0.023751
13	36698.8	272.5	0.059385690	H - 4 → L<br>H - 3 → L<br>H - 1 → L + 3<br>H - 1 → L + 4<br>H → L + 4<br>H → L + 5<br>H → L + 6<br>H → L + 7	0.043926<br>0.615651<br>0.011584<br>0.018208<br>0.060437<br>0.131002<br>0.015664<br>0.063258
14	37110.1	269.5	0.018791604	H - 4 → L<br>H - 3 → L<br>H - 2 → L + 3	0.011776<br>0.243732<br>0.019335

				H - 1 → L + 4<br>H → L + 4<br>H → L + 5<br>H → L + 6	0.012286<br>0.034512<br>0.575168<br>0.065536
15	37472.6	266.9	0.005185902	H - 2 → L + 3<br>H - 1 → L + 4<br>H → L + 5<br>H → L + 6	0.234712<br>0.650051<br>0.023363<br>0.041121
16	37774.6	264.7	0.045871278	H - 5 → L<br>H - 4 → L<br>H - 3 → L<br>H - 2 → L + 3<br>H → L + 5<br>H → L + 7	0.012486<br>0.678975<br>0.067748<br>0.051018<br>0.028604<br>0.107901
17	38012.9	263.1	0.015403972	H - 4 → L<br>H - 2 → L + 3<br>H - 2 → L + 4<br>H - 1 → L + 3<br>H - 1 → L + 4<br>H - 1 → L + 5<br>H → L + 3<br>H → L + 5<br>H → L + 6	0.037673<br>0.527308<br>0.050422<br>0.055942<br>0.167187<br>0.021845<br>0.011205<br>0.024761<br>0.070120
18	38669.4	258.6	0.006293214	H - 2 → L + 3<br>H - 2 → L + 4<br>H - 1 → L + 4<br>H - 1 → L + 5<br>H → L + 6	0.024066<br>0.352542<br>0.020576<br>0.275696<br>0.290604
19	38747.7	258.1	0.000449211	H - 5 → L<br>H - 4 → L	0.957471<br>0.027537
20	38917.9	257.0	0.011894574	H - 2 → L + 3<br>H - 2 → L + 4<br>H - 1 → L + 5<br>H - 1 → L + 6<br>H → L + 4<br>H → L + 5<br>H → L + 6	0.012426<br>0.532882<br>0.106275<br>0.029428<br>0.023167<br>0.035634<br>0.228919

Frontier orbitals

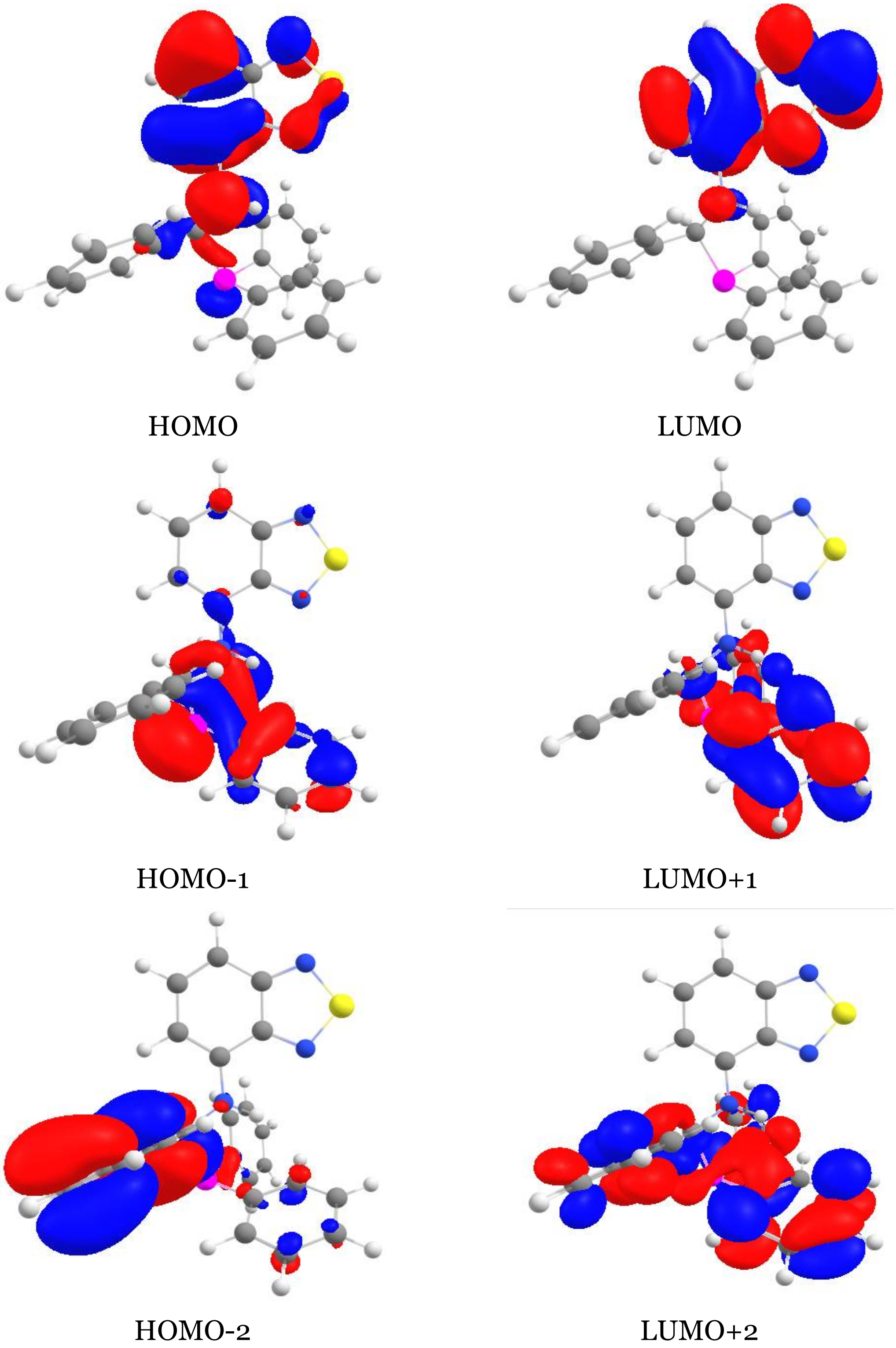

Figure S8. Frontier orbitals of **1** in the ground state geometry (PBEO/def2-TZVP(-f), isosurface = 0.03)

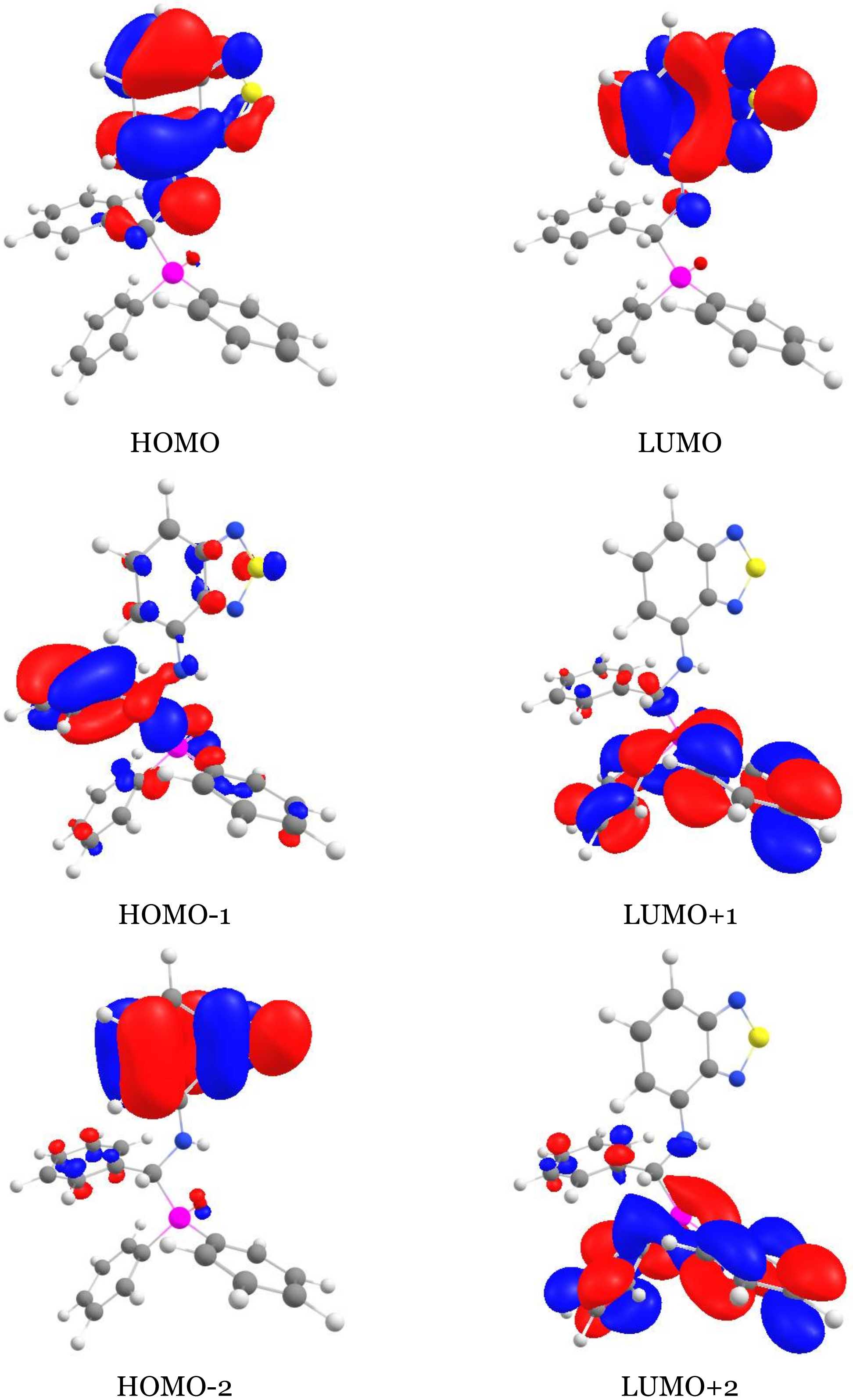

Figure S9. Frontier orbitals of **2** in ground state geometry (PBEO/def2-TZVP(-f), isosurface = 0.03)

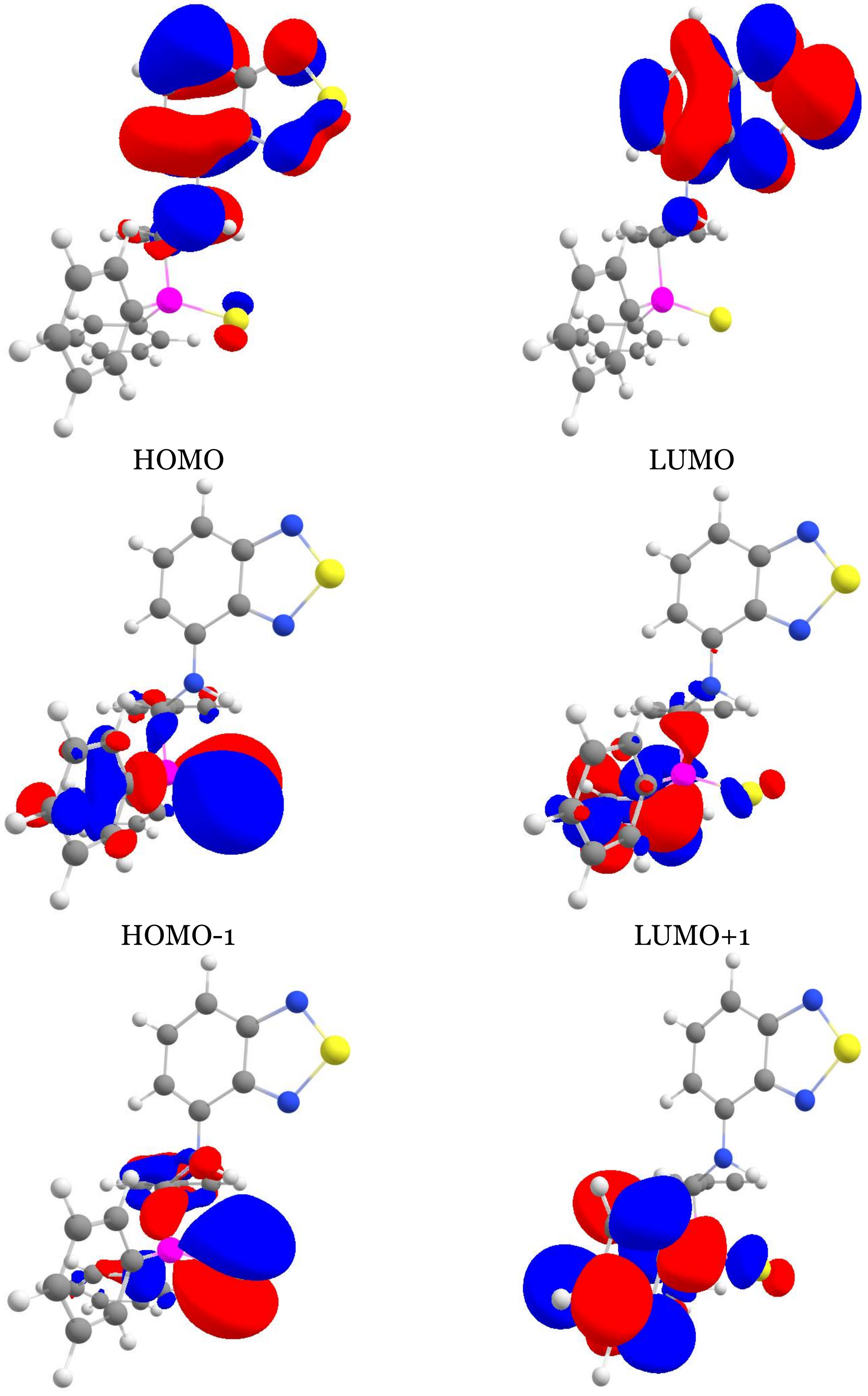

Figure S10. Frontier orbitals of **3** in ground state geometry (PBE0/def2-TZVP(-f), isosurface = 0.03)

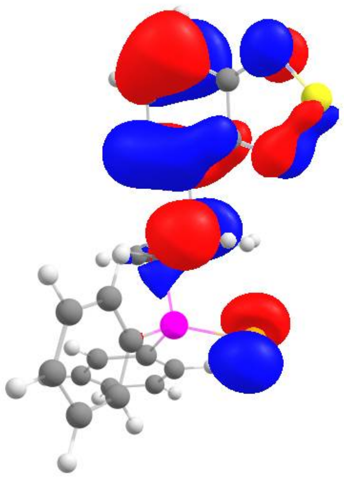

HOMO

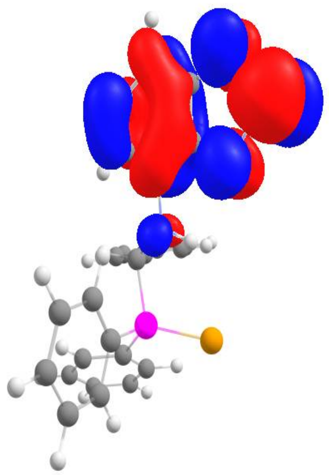

LUMO

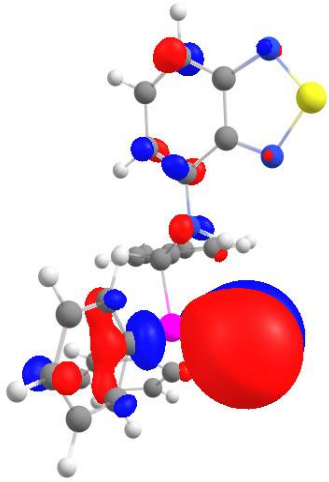

HOMO-1

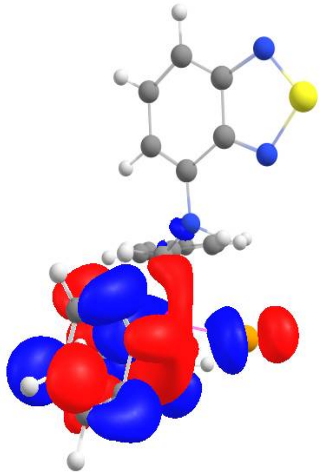

LUMO+1

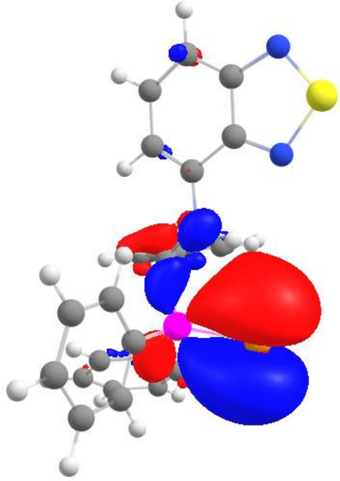

HOMO-2

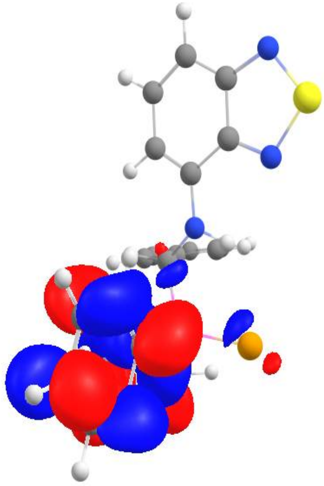

LUMO+2

Figure S11. Frontier orbitals of **4** in ground state geometry (PBEO/def2-TZVP(-f), isosurface = 0.03)

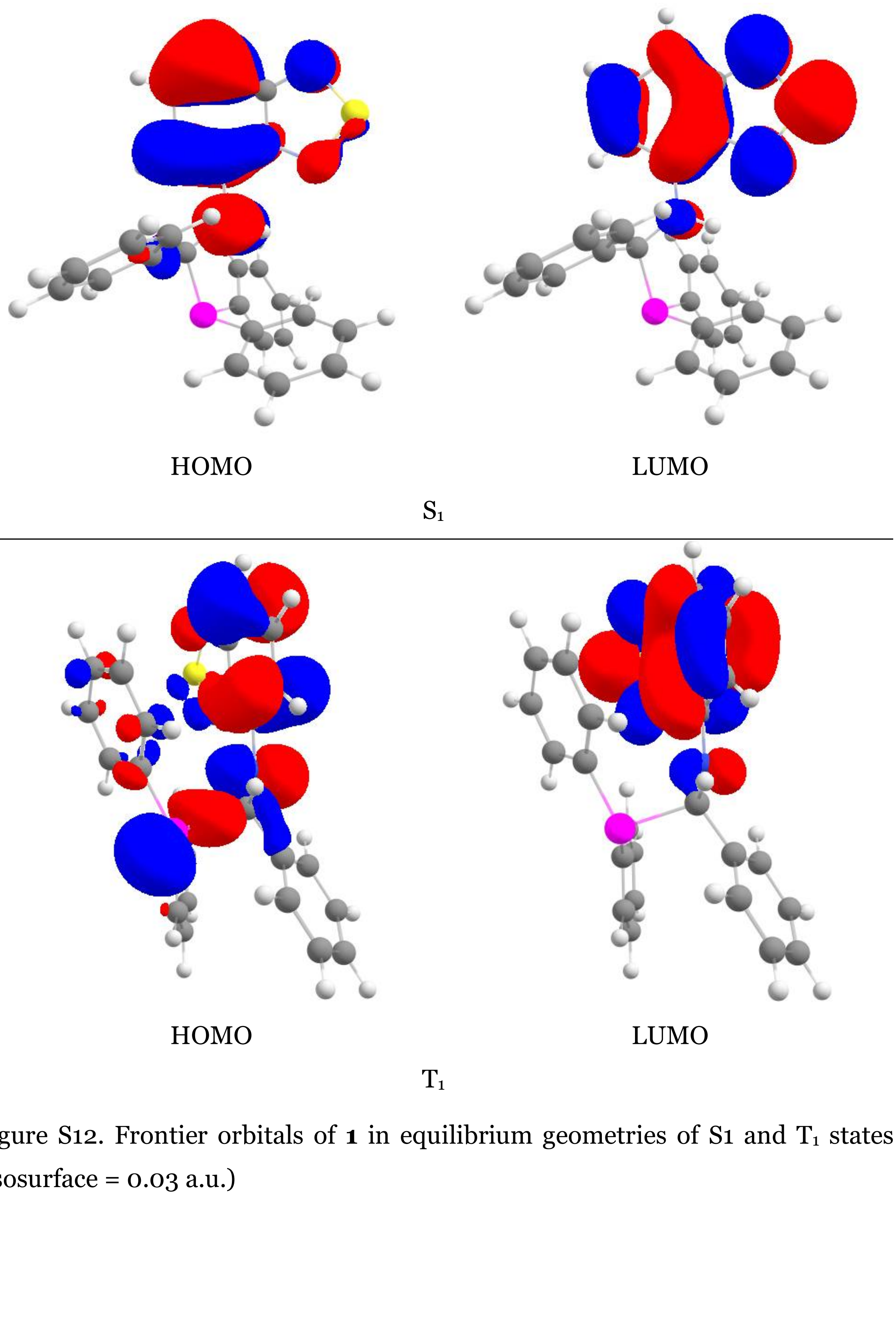

Figure S12. Frontier orbitals of **1** in equilibrium geometries of S1 and T_1 states (isosurface = 0.03 a.u.)

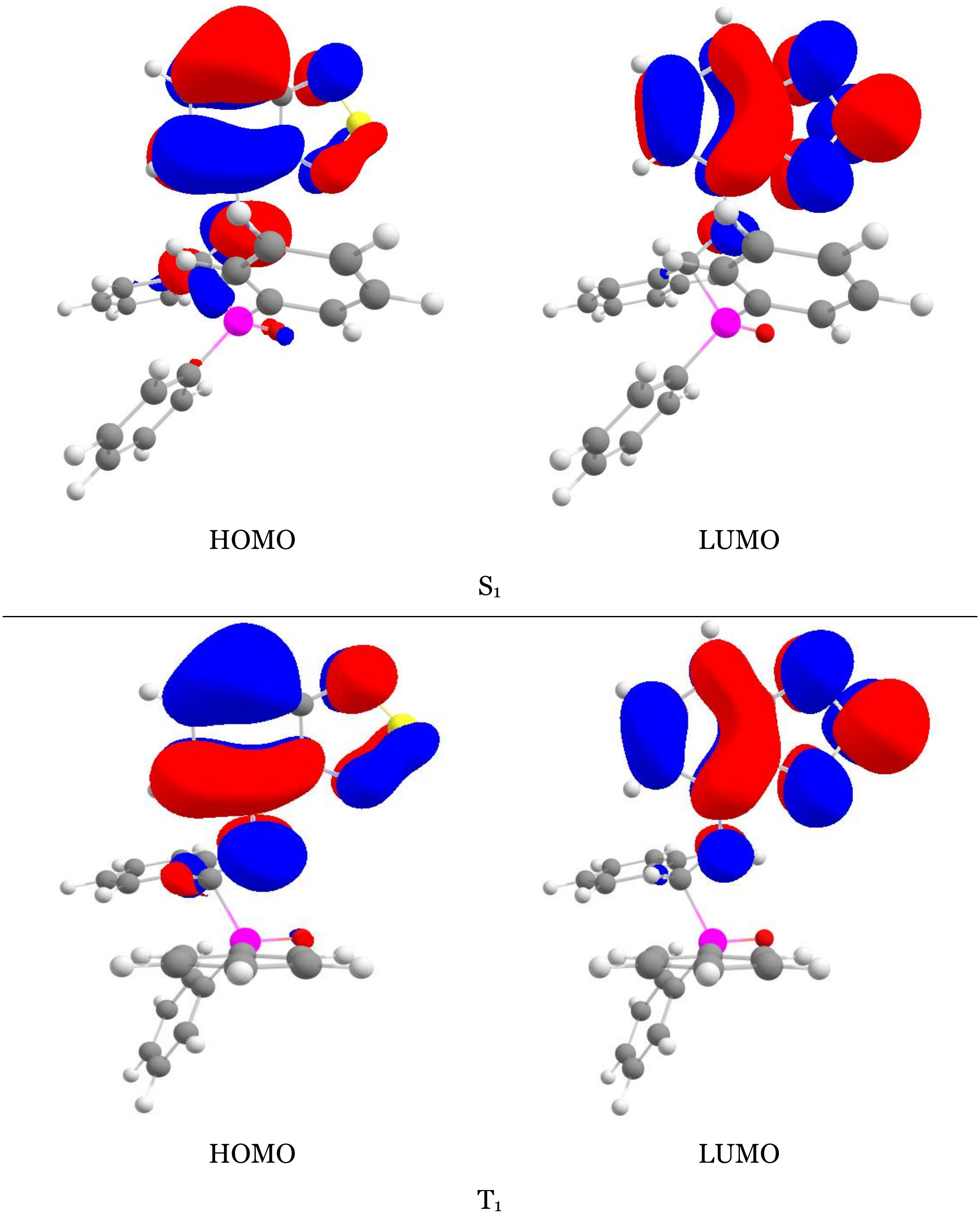

Figure S13. Frontier orbitals of **2** in equilibrium geometries of S1 and T_1 states (isosurface = 0.03 a.u.)

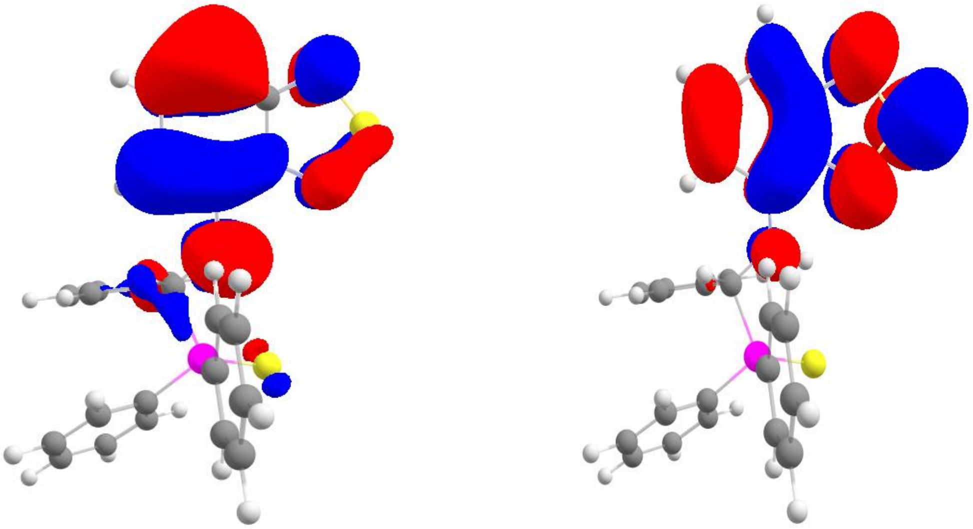

HOMO LUMO

S_1

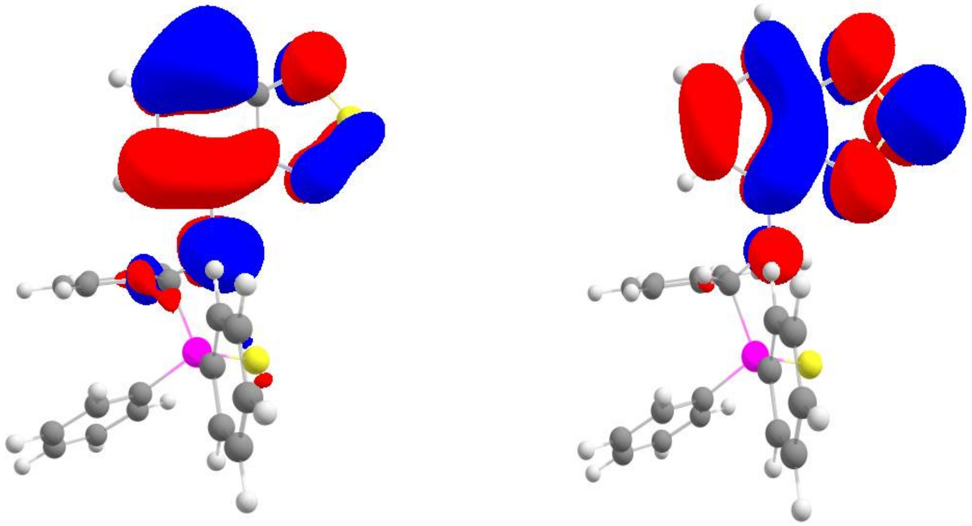

HOMO LUMO

T_1

Figure S14. Frontier orbitals of **3** in equilibrium geometries of S1 and T_1 states (isosurface = 0.03 a.u.)

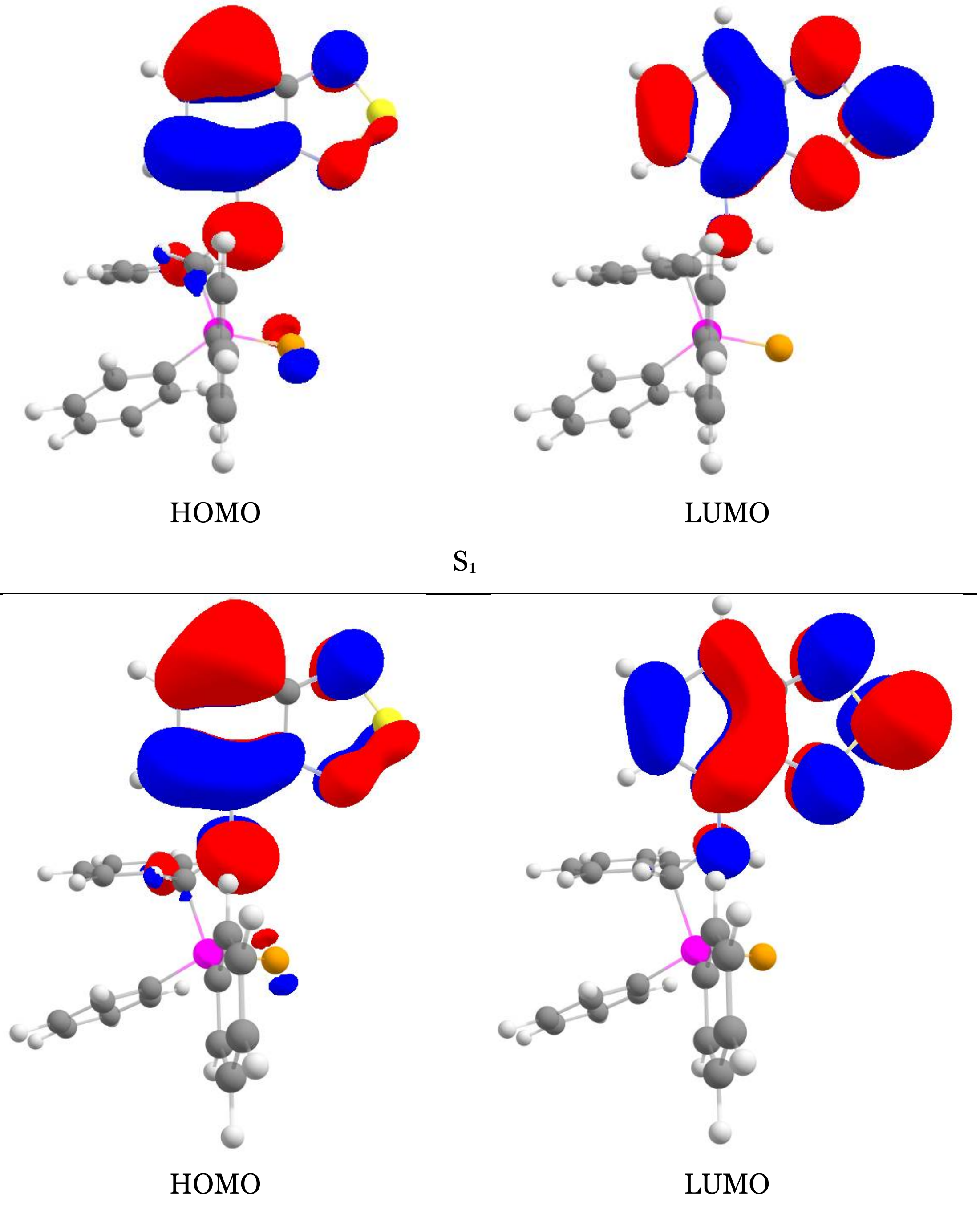

Figure S15. Frontier orbitals of **4** in equilibrium geometries of S1 and T_1 states (isosurface = 0.03 a.u.)

DFT calculation of relaxation processes

Table S7. 0-0 transition wavelengths and energies, calculated by PBE0/def2-TZVP(-f) over equilibrium excited state geometries.

Compound	S_1	T_1
**1**	528.2 nm (2.347 eV)	904.3 (1.371 eV)
**2**	519.0 nm (2.389 eV)	821.2 (1.510 eV)
**3**	509.6 nm (2.433 eV)	821.4 nm (1.510 eV)
**4**	508.1 nm (2.440 eV)	819.8 nm (1.512 eV)

Table S8. Selected geometric parameters of the molecules calculated in S1 and T1 excited states

	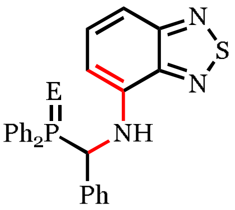	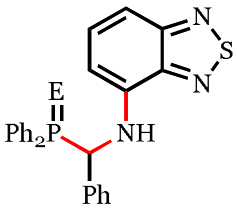	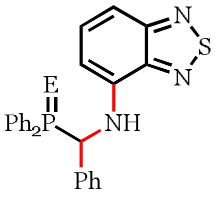	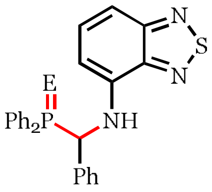
	CCNC, deg	CNCP, deg	CNCC, deg	NCPE, deg
		**1**		
S1	8.79	166.51	71.36	-
T1	38.38	94.21	143.36	-
		**2**		
S1	8.60	140.94	98.04	51.81
T1	3.43	155.84	77.29	34.11
		**3**		
S1	1.99	160.32	77.49	44.85
T1	0.19	164.08	73.52	45.84
		**4**		
S1	2.39	159.90	77.40	44.59
T1	0.48	163.65	73.60	44.64

Photoluminescence spectra

	77 K	300 K
2 α	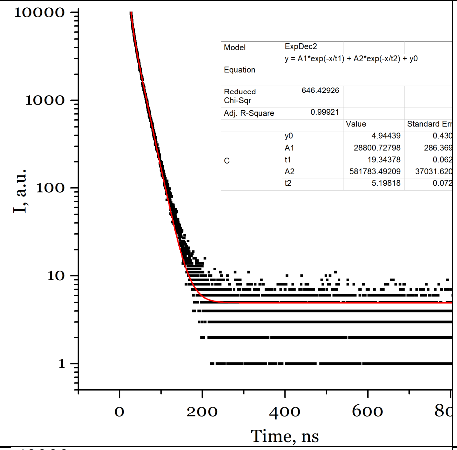	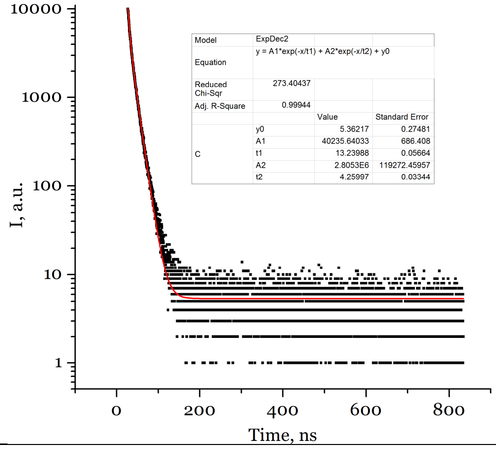
3	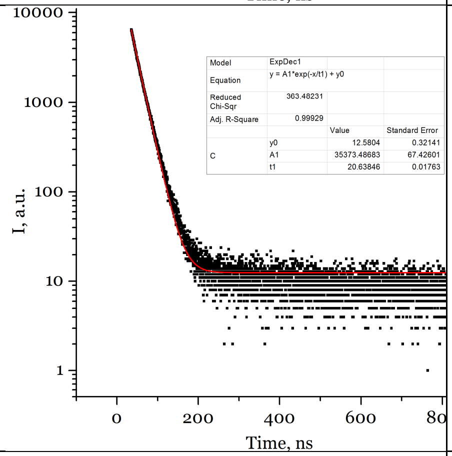	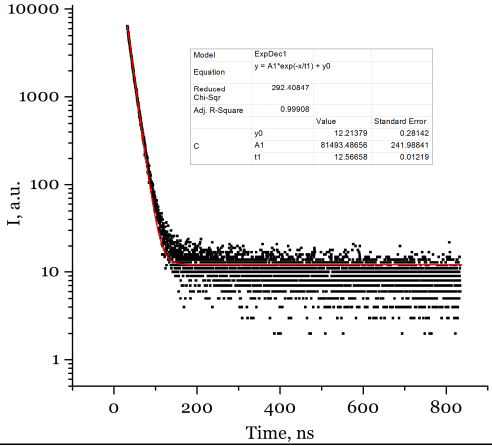
4	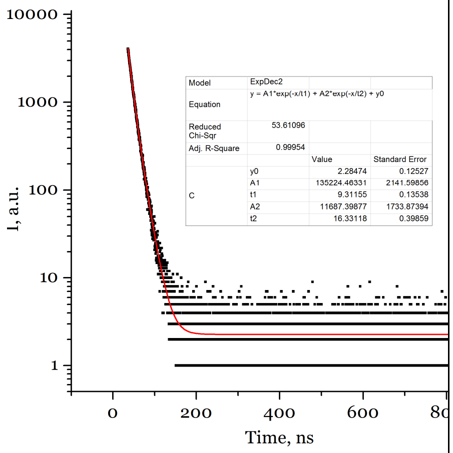	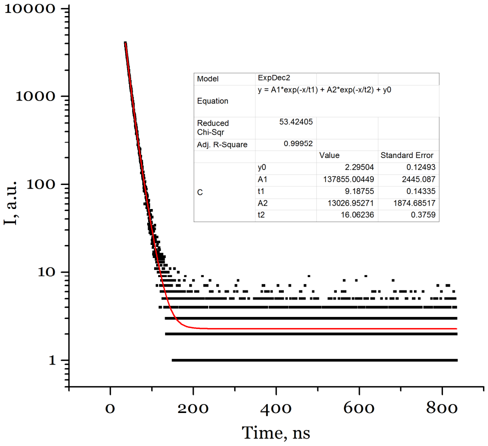

Figure S16. Emission decay kinetics of the compounds at 77 and 300 K. The fit is shown as red line.

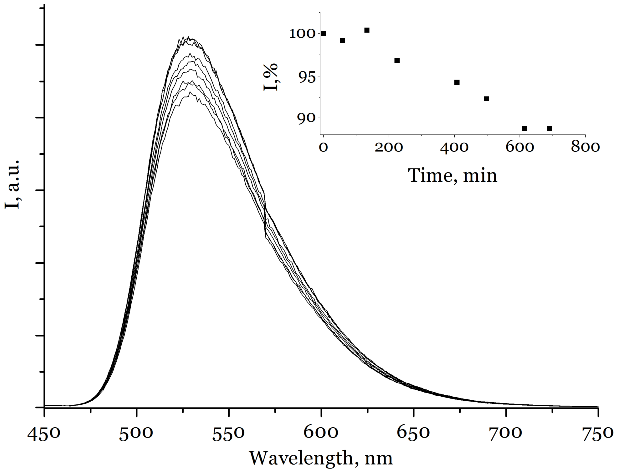

Figure S17. PL spectra of compound **3** upon continuous irradiation with a visible light on air. Inset: the emission intensity at 530 nm normalized on 100% vs irradiation time.

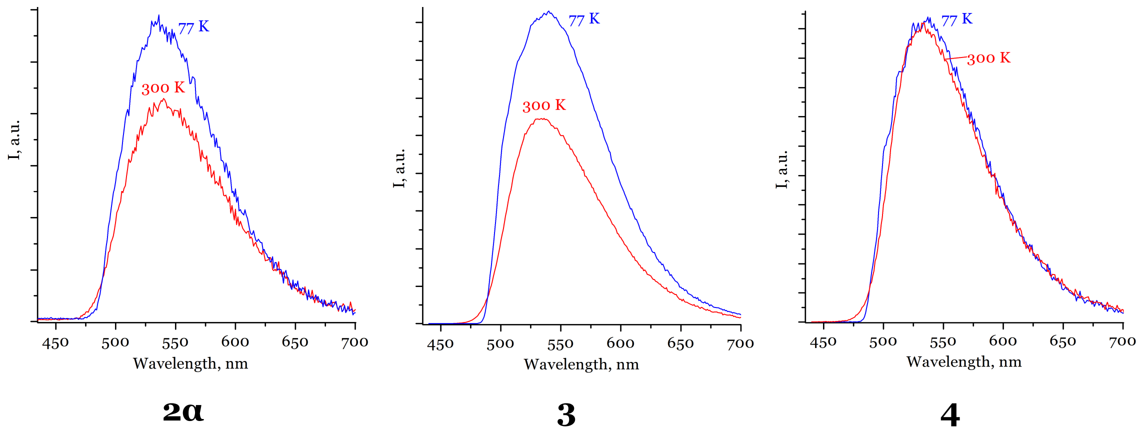

Figure S18. Comparison of the PL spectra for the compounds at 77 and 300K.

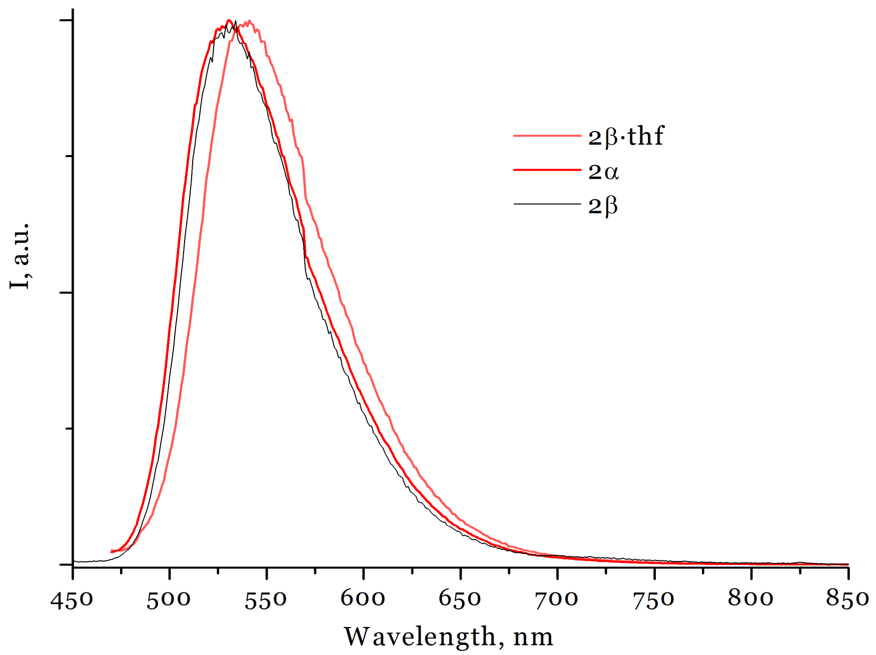

Figure S19. Comparison of the normalized PL spectra for compounds **2α**, as-synthesized **2β**·thf, and unsolvated **2β**.

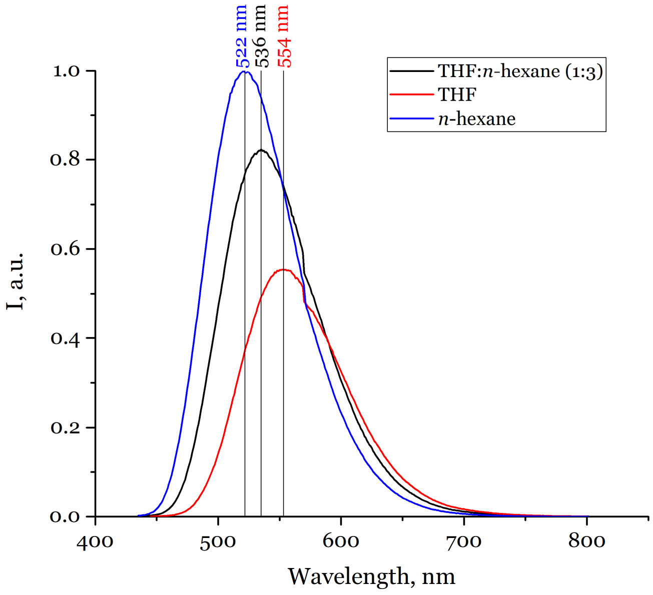

Figure S20. PL spectra of **1** in THF, *n*-hexane and mixture (THF:*n*-hexane = 1 : 3), intensities normalized to relative to spectrum of *n*-hexane solution

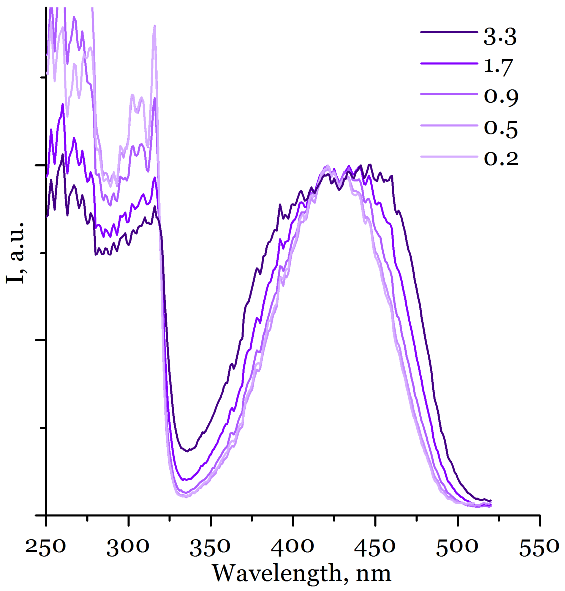

Figure S21. Excitation spectra at 520 nm of polystyrene films containing **2** with a mass fraction from 0.2 to 3.3%, normalized on the long wavelength band.
